# Regional Differences in Height, Weight, and Body Composition may Result from Photoperiodic Responses: An Ecological Analysis of Japanese Children and Adolescents

**DOI:** 10.5334/jcr.198

**Published:** 2021-02-22

**Authors:** Masana Yokoya, Aki Terada

**Affiliations:** 1 Shimonoseki Junior College, Shimonoseki, Yamaguchi 750-8508, Japan

**Keywords:** Obesity, Day length, Geographical difference, Growth seasonality, Epigenetics, Photoperiodic history

## Abstract

This ecological study examined whether geographical differences in the physique of Japanese children and adolescents can be explained from the perspective of photoperiodicity induced by effective day length (light duration exceeding a certain threshold of illuminance) using prefecture-level anatomical data and Mesh Climatic Data. Multiple regression analysis for height prediction demonstrated that when controlled by weight, effective day lengths of the longest and shortest months were inversely correlated with height distribution. Conversely, for weight prediction, when controlled by height, the effective day lengths of the longest and shortest months were positively correlated with weight distribution. The regression coefficients were greater for the effective day length of the shortest month in both height and weight prediction. This phenomenon where the same two explanatory variables are negatively correlated with height and positively correlated with weight in a significant manner is rare, and there may be no physiological interpretation of this phenomenon other than one based on changes in thyroid hormone signaling. These distribution characteristics are common to the photoperiodicity by which seasonal breeding vertebrates reciprocally switch thyroid hormone signaling according to prior photoperiodic history through epigenetic functions. From these perspectives, thyroid hormone signaling in a certain region was assumed to be activated in summer according to the prior shorter winter day length and inactivated in winter according to the prior longer summer day length. Regarding the prevalence of obesity, the coexistence of longer summer and winter day lengths was thought to set body composition to be short and fat in early adolescence.

## Introduction

The physique of Japanese children tends to increase at higher latitudes and has a north-south gradient [1,2,3,4,5]. This trend has continued for at least 50 years, during which the national average of children’s physique has increased. However, the north-south gradient remains almost unchanged. After consideration of the improvement of nutrient intake, hygienic environment, and intense migration that occurred in the last 50 years throughout Japan, the north-south gradient in physique is probably the result of environmental rather than nutritional or genetic factors [1,5].

The authors previously reported that geographical differences in effective day length may cause differences in thyroid hormone signaling in the endocrine system, resulting in geographical differences in physique [5,6,7]. The effective day length is the time for which the light intensity is considered as it indicates the duration of light exceeding a certain threshold of illuminance [7].

In a previous ecological study using prefectural-level anatomical data and climatic data, the multiple regression analysis demonstrated that the combination of annual mean effective day length and weight was statistically significant as a predictor of height. Controlling for bodyweight revealed that effective day length was inversely correlated with height. Conversely, the multiple regression analysis demonstrated that a combination of annual mean effective day length and height was statistically significant as a predictor of weight. Controlling for height revealed that effective day length was positively correlated with weight. In short, the effective day length seems to affect both height and weight increase; however, the direction of the effect is the opposite. Although these characteristics of distribution appear to be inconsistent, it can be explained by assuming that it is the effect of thyroid hormone metabolism [5]. Thyroid hormone is an essential hormone for the growth of children and adolescents, with hyperthyroidism known to cause a tall and thin body composition [8,9], and hypothyroidism known to cause a short and fat body composition [10,11].

Many similarities exist between the characteristics of these distributions and the photoperiodicity of seasonal breeding vertebrates. Seasonal breeding vertebrates are known to reciprocally switch hypothalamic thyroid hormone signaling according to the photoperiodic environment through epigenetic function. Two deiodinase enzyme genes (*DIO2* and *DIO3*) are regulated to determine the local concentration of the biologically active form of thyroid hormone, triiodothyronine (T3) [12,13,14,15]. These signals are transmitted to further downstream reactions, which are known to influence reproduction and immune seasonality. The switching mechanism of thyroid hormone signaling is highly conserved among vertebrates [16].

Assuming that human growth is affected by the photoperiodic environment in the same way as seasonal breeding vertebrates, and reaches sexual maturity by repeated acceleration and deceleration of seasonal development over several years, the relationship between geographical differences in physique and day length can be well explained [5].

In a previous ecological study, the height of Japanese children (controlled with weight) negatively correlated with the annual mean effective day length, and the authors assumed that thyroid hormone signaling was activated in the region with shorter effective day length. In contrast, the weight of Japanese children (controlled with height) positively correlated with the annual mean effective day length, and the authors assumed that thyroid hormone signaling was inactivated in the region with longer effective day length. However, there is not yet sufficient evidence to suggest that regional differences in physique occur as photoperiodicity.

The photoperiodic response is not an immediate response to changes in day length, but rather anticipated responsiveness to ensure that physiological changes occur at the appropriate time [14]. For example, seasonal breeding vertebrates such as Siberian hamsters perform reproductive activities in anticipation of the arrival of spring according to shorter winter day length [17]. If these geographical differences in physique are due to the photoperiodic response of seasonal acceleration and deceleration, physical development in an area should occur as an anticipated reaction. In a previous report, we noted that although height is more likely gained in the long-day season, height tends to increase in the region with shorter day length, suggesting that the height increase velocity in summer may be greater according to the shortness of the winter day length. If these geographical differences in physique are due to the photoperiodic response in the biological sense, they should be described by summer day length (longest day length) and winter day length (shortest day length) rather than by yearly averages.

Furthermore, the results of the previous report suggest that differences in the photoperiodic environment lead to more obesity in certain areas. These results indicate that if the weight remains constant, as the day length increases, height decreases; if the height remains constant, as the day length increases, weight increases. This means that short and fat body composition is more likely to occur in certain areas due to the geographical difference in effective day length [5]. However, in reality, if the effective day length is short, the physique tends to be small and obesity does not increase. If we know the annual mean effective day length, we can forecast body size; however, we cannot predict body composition [5]. This may be because the annual mean effective day length is an index of the average environmental daylight and lacks the perspective of seasonality. Again, there is a possibility that the geographical differences in body composition and region-specific prevalence of obesity can be explained by the relevance of summer day lengths and winter day lengths. Obesity is often said to be region-specific; however, the cause is largely unknown [18,19,20,21,22]. The regional specificity of obesity may be explained by geographical differences in the photoperiodic environment.

Therefore, in this study, we considered whether the geographical difference in body height, weight, and body composition (obesity) could be described based on geographical differences in summer and winter effective day lengths instead of yearly averages, using precise climatic data and the Geographic Information Science (GIS) technique. Additionally, we investigated whether physiological mechanisms can be explained from the perspective of photoperiodicity.

## Materials and Methods

### Study area

This ecological study was conducted using prefectural-level data from Japanese children and adolescents aged between 5 and 17 years. S1 Fig shows a map of 47 prefectures in Japan. The displayed prefecture codes are the same as those in S1–S4 Tables [5].

### Anatomical data

Prefectural-level anatomical data of Japanese children and adolescents were collected from the School Health Examination Survey conducted by the Ministry of Education, Culture, Sports, Science, and Technology between 1989 and 2013 [23,24]. These surveys cover each of the 47 prefectures in Japan and include data on average height, weight, and other physical conditions classified according to sex and age (5 to 17 years). Sample sizes and original profiles have been published in this database [23,24].

To obtain stable data and to focus on geographical differences, a standardized 25-year average of height and weight (1989–2013) was calculated for each sex and age category:

1 {Y_{ij}} = \frac{1}{{25}}\sum\nolimits_{k = 1}^{25} {\frac{{{Z_{ijk}} - {u_{jk}}}}{{{\sigma _{jk}}}}}

where *i* is the prefecture, *j* is the group (defined by age and sex), *k* is the year (1989–2013), *Y_ij_* is the standardized data over the 25-year period for each prefecture and sex standardized by mean, *Z_ij_* represents the prefecture data, *μ_jk_* is the national mean, and *σ_jk_* is the national-level standard deviation based on the entire set of measurements obtained from the survey reports [23].

The standardized heights and weights averaged for the 25-year period for each prefecture are listed in Table S1 and S2. The association between standardized height and weight is listed in S3 Table [5].

### Climatic data

In previous studies, the concept of effective day length was proposed as a climatic factor to quantify the intensity and duration of ambient sunlight [7]. Effective day length is the photoperiod taking into account the light intensity. The effective day length increases as the amount of solar radiation increases. The effective day length for which the illumination threshold is greater than 0 lux is the same as the possible sunshine duration hours. The effective day length at more than 1000 lux is almost directly proportional to the amount of solar radiation in the data range observed in Japan, and any effective day length is almost directly proportional to another above the threshold of 1000 lux. The monthly means of effective day length at any light threshold can be calculated by the empirical model using a monthly amount of solar radiation data [5,7].

Mesh Climatic Data 2000 [25] was used to compare the geographical distribution of height and weight. Mesh Climatic Data 2000 is a map of climatological normal that represents the average climate over 30 years from 1971 to 2000 and was developed by extending observed data from Japanese meteorological stations in consideration of topographical factors. Mesh Climatic Data 2000 contains data such as the monthly amount of solar radiation with a grid resolution of 1 km^2^. The monthly amount of solar radiation data was used as an input value to calculate the monthly value of the effective day length [5].

Since any threshold of effective day length calculated on a prefectural basis is almost directly proportional to another at its threshold exceeding 1000 lux, 5000 lux was set as a convenient threshold for the effective day length in this analysis. Because the distribution of solar radiation and any threshold of effective day length exceeding 1000 lux is almost proportional to each other, any threshold of effective day length exceeding 1000 lux becomes longer in regions receiving greater amounts of solar radiation. In conclusion, since any threshold of effective day length exceeding 1000 lux will increase in regions receiving greater solar radiation, residents in those areas will be exposed to bright daylight for longer periods [5].

When considered at the individual level, the threshold of intensity of natural daylight affecting the person should differ depending on the house structure, bedroom shape, window size, and presence of curtain in the person’s house as well as the person’s lifestyle. However, such differences at the individual level are considered to be an offset in population-based studies [5].

Because the population varies from mesh to mesh, it is not desirable to obtain the prefectural level effective day length based on a simple average of climatic mesh values. To account for spatial differences between populations, Mesh Population Data compiled from the results of the 2005 Population Census [26] and produced under the same code and standards as the Mesh Climatic Data 2000 were used to calculate the population-weighted prefectural mean effective day length (*Eavg*) for each prefecture using the following formula:

2 Eavg = \frac{{\mathop \sum \nolimits_{n = 1}^m \left( {T \times P} \right)}}{{\mathop \sum \nolimits_{n = 1}^m \left( P \right)}}

where *m* is the number of meshes in each prefecture, *T* is the effective day length in the mesh, and *P* is the population density of the mesh [5].

To focus on the seasonality of day length, effective day length of the longest month and effective day length of the shortest month were extracted for each prefecture. These data were used as explanatory variables for the multiple regression analysis. We listed the population-weighted effective day length of the longest and shortest months at 5000 lux derived from the mesh data of each prefecture in S4 Table [5].

### Data analysis

Correlation analysis was performed using standardized height and weight data and population-weighted climatic data (effective day length of the longest and the shortest months) for all 47 prefectures. The association between anatomical data and climatic data was further examined via multiple linear regression modeling.

Previous ecological studies have reported that the following formulas hold simultaneously for height, weight, and annual mean effective day length. The distribution of the physique of Japanese early adolescence can be well described by the following two formulas [5].

3 {H_i} = {k_1}{W_i} - {k_2}{E_i},

4 {W_i} = {k_1}{H_i} + {k_2}{E_i},

where *H* is the linear predictor of the mean height for each prefecture in area *i, W* is the mean weight for each prefecture in area *i, E* is the population-weighted annual mean effective day length in area *i*, and *k*_1_ and *k*_2_ are the standardized regression coefficients. In this model, annual mean effective day length was a negative predictor of height and a positive predictor of weight.

In this study, the effective day lengths of the longest and shortest months were used as explanatory variables, instead of the annual mean effective day length.

5 {H_i} = {k_1}{W_i} - {k_2}LON{G_i} - {k_3}SHOR{T_i}

6 {W_i} = {k_1}{H_i} + {k_2}LON{G_i} + {k_3}SHOR{T_i}

where *H* is the linear predictor of the mean height for each prefecture in area *i, W* is the mean weight for each prefecture in area *i, LONG* is the population-weighted effective day length of the longest month in area *i, SHORT* is the population-weighted effective day length of the shortest month in area *i*, and *k*_1_, *k*_2_, and *k*_3_ are the standardized regression coefficients. Both multiple regression models (5) and (6) were applied to boys and girls aged 5 to 17 years. Furthermore, based on the results of the multiple regression model, we tried to explain the cause of the region-specific differences in the prevalence of short and fat body composition (obesity) from the viewpoint of seasonal differences in effective day length.

All statistical analyses were performed using R version 3.5.3 [27].

## Results

**Figure 1:**
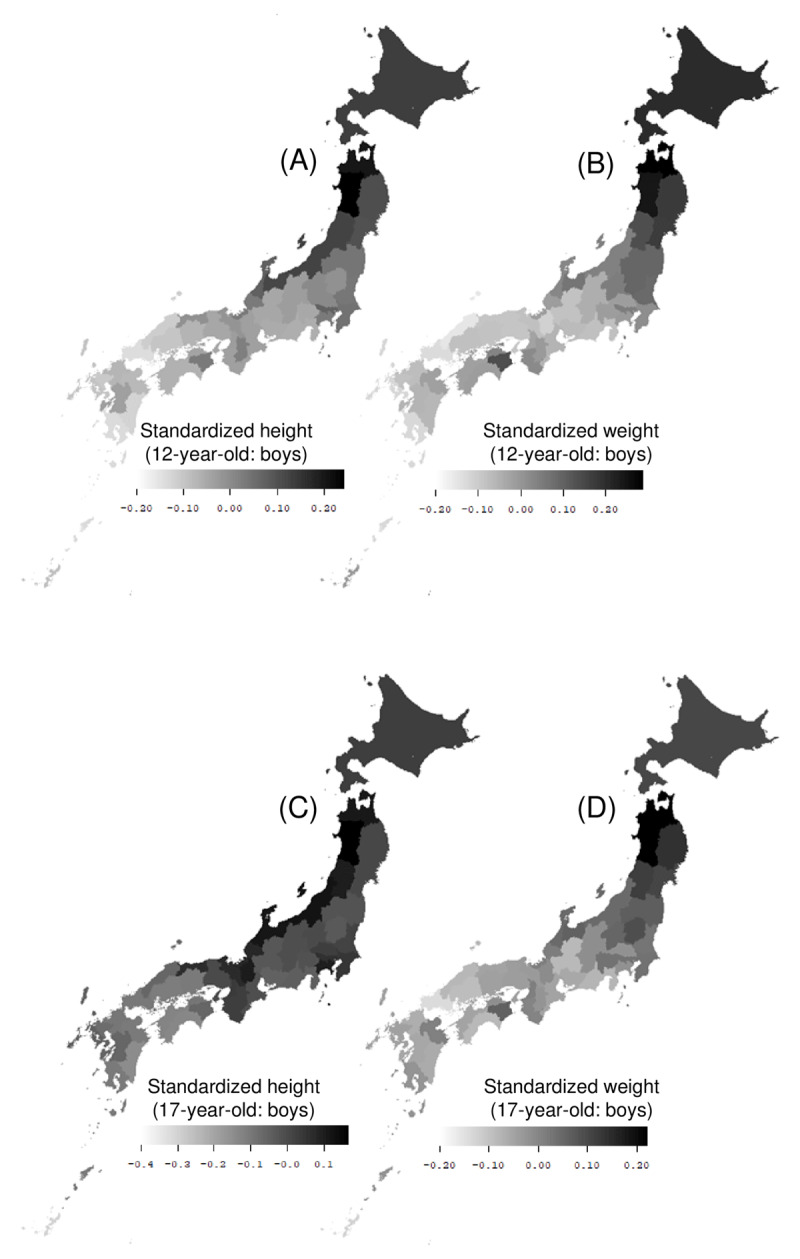
**Map of the distribution of standardized heights and weights of Japanese youth.** Map of the distribution of the 25-year (1989–2013) average of standardized heights and weights of the following subject groups in each prefecture: **(A)** heights of 12-year-old boys, **(B)** weights of 12-year-old boys, **(C)** heights of 17-year-old boys, and **(D)** weights of 17-year-old boys [5,6].

Figure 1 shows a map of the standardized height and weight distribution of 12 and 17-year-old Japanese boys over a 25-year study period. The height tends to be greater in northern Japan and northern areas along the coast of Japan. In addition, in northern Japan, the weight tends to increase. However, the weight distribution displays a band-like pattern (parallel to the latitude) [5].

Table 1 shows the basic statistics on the height and weight of Japanese children and adolescents standardized over 25 years from 1989 to 2013. Maximum heights were observed in northern Japan (Akita, Aomori), while minimum heights were observed in southern Japan (Okinawa, Miyazaki, and Yamaguchi). The maximum weights were observed in northern Japan (Aomori and Akita), whereas the minimum weights were observed in southern Japan (Okinawa, Yamaguchi, and Shimane) [5].

**Table 1:** Basic statistics for standardized heights and weights.

Standardized Height										

	Boys					Girls				

Age	5	8	11	14	17	5	8	11	14	17
Maximum	0.214	0.218	0.237	0.219	0.166	0.221	0.223	0.226	0.158	0.121
	Akita	Akita	Akita	Akita	Akita	Akita	Aomori	Aomori	Akita	Akita
Minimum	–0.261	–0.246	–0.135	–0.171	–0.305	–0.202	–0.133	–0.100	–0.306	–0.323
	Okinawa	Okinawa	Yamaguchi	Miyazaki	Okinawa	Okinawa	Okinawa	Yamaguchi	Okinawa	Okinawa
Mean	–0.009	–0.006	–0.001	–0.008	–0.011	–0.003	0.001	0.004	–0.012	–0.013
Median	–0.014	0.000	–0.028	–0.018	–0.008	–0.010	–0.007	–0.006	–0.020	–0.007
**Standardized Weight**										

	**Boys**					**Girls**				

Age	5	8	11	14	17	5	8	11	14	17
Maximum	0.234	0.295	0.289	0.262	0.220	0.242	0.293	0.255	0.229	0.195
	Aomori	Aomori	Aomori	Aomori	Akita	Aomori	Aomori	Aomori	Aomori	Akita
Minimum	–0.133	–0.106	–0.157	–0.158	–0.139	–0.135	–0.099	–0.099	–0.107	–0.254
	Okinawa	Yamaguchi	Shimane	Yamaguchi	Yamaguchi	Okinawa	Yamaguchi	Yamaguchi	Okinawa	Okinawa
Mean	–0.001	0.008	0.003	–0.002	0.002	0.003	0.013	0.011	0.011	0.014
Median	–0.031	–0.026	–0.030	–0.025	–0.016	–0.031	–0.014	–0.008	–0.015	0.002

**Figure 2:**
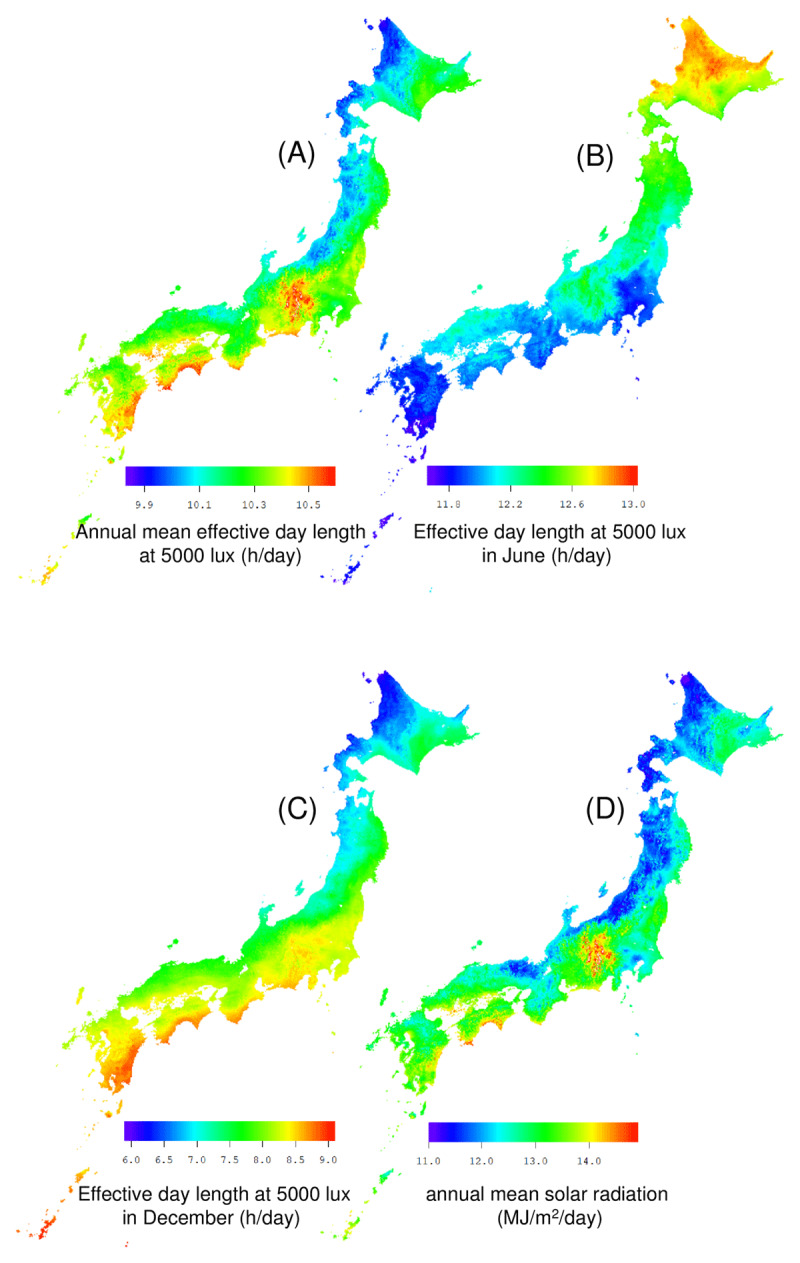
**Distribution map of solar radiation and effective day length at 5000 lux in Japan.** The fill in the mesh areas indicates **(A)** annual mean effective day length at 5000 lux (h/day), **(B)** effective day length at 5000 lux in June (h/day), **(C)** effective day length at 5000 lux in December (h/day), and **(D)** annual mean solar radiation (MJ/m^2^/day) [5,6].

Figure 2 shows the distribution of the annual mean solar radiation and effective day length at 5000 lux in Japan, extracted from the Japanese Mesh Climatic Data 2000 for 380,000 one km^2^ mesh areas. The effective day length at 5000 lux was estimated using the empirical model [7]. The filled-in mesh areas indicates (A) annual mean effective day length at 5000 lux (h/day), (B) effective day length at 5000 lux in June (h/day), (C) effective day length at 5000 lux in December (h/day), (D) annual mean solar radiation (MJ/m^2^/day) [5,6]. Since the month in which the effective day length becomes the longest or the shortest is different depending on the prefecture, the distributions of June and December are shown for convenience. More than half of the prefectures have their longest effective day length in June and their shortest effective day length in December (S4 Table). Annual mean solar radiation, annual mean effective day length, and effective day length in December tend to be relatively low in the northern areas along the coast of Japan. Effective day length in June tends to be longer in Hokkaido, and shorter around the Kanto region (around Tokyo) and the Kyushu region (southwestern Japan).

Table 2 shows the basic statistics of the 30-year average of the population-weighted effective day length at 5000 lux. The maximum effective day length of the longest month (summer) was observed in Hokkaido, and the minimum was observed in Tokyo and Kanagawa. The possible sunshine duration in summer increases in the northern regions of Japan. However, the southern region has large amounts of solar radiation in summer. In the vicinity of Japan, this effect is offset, and the effective day length of the longest month is shortest near Tokyo. The maximum effective day length of the shortest month (winter) was observed in Okinawa and the minimum in Akita. The data range of the shortest effective day length was longer than that of the longest.

**Table 2:** Basic statistics for climatic variables.

	Effective day length of the longest month at 5000 lux (h/day)	Effective day length of the shortest month at 5000 lux (h/day)

Maximum	12.6 (Hokkaido: Jun)	8.9 (Okinawa: Jan)
Minimum	11.8 (Tokyo: May–July, Kanagawa: May–July)	6.9 (Akita: Dec)
Mean	12.02	8.08
Median	12.00	8.30

Table 3 shows Pearson’s correlation coefficient for the relationship between height, weight, effective day length of the longest and shortest months at 5000 lux. Both height and weight were positively correlated with the effective day length of the longest month (summer) and negatively correlated with the effective day length of the shortest month (winter). This tendency was the same in all categories of boys and girls aged 5–17. The effective day length of the longest month tended to be more strongly correlated with weight than height. The effective day length of the shortest month was more strongly correlated with height than weight. The correlation coefficients between height and weight reached a peak in early adolescence.

**Table 3:** Pearson’s correlation coefficients for the relationship between height, weight, and effective day length of the longest or shortest months.

	Height vs. effective day length of the longest month	Height vs. effective day length of the shortest month	Weight vs. effective day length of the longest month	Weight vs. effective day length of the shortest month	Height vs. weight

Boys					
Age: 5	0.54**	–0.80**	0.53*	–0.58**	0.82**
6	0.50*	–0.75**	0.58**	–0.62**	0.82**
7	0.54*	–0.79**	0.65**	–0.68**	0.82**
8	0.53*	–0.80**	0.65**	–0.67**	0.81**
9	0.54**	–0.81**	0.66**	–0.68**	0.83**
10	0.59**	–0.82**	0.65**	–0.64**	0.86**
11	0.61**	–0.81**	0.63**	–0.61**	0.88**
12	0.64**	–0.81**	0.65**	–0.60**	0.87**
13	0.61**	–0.84**	0.65**	–0.62**	0.84**
14	0.52*	–0.83**	0.64**	–0.65**	0.78**
15	0.41*	–0.79**	0.57**	–0.64**	0.68**
16	0.36	–0.76**	0.58**	–0.68**	0.66**
17	0.33	–0.74**	0.62**	–0.71**	0.63**
Girls					
Age: 5	0.57**	–0.79**	0.52*	–0.57**	0.84**
6	0.55**	–0.76**	0.59**	–0.62**	0.84**
7	0.56**	–0.81**	0.62**	–0.66**	0.83**
8	0.58**	–0.82**	0.63**	–0.66**	0.85**
9	0.61**	–0.82**	0.65**	–0.64**	0.86**
10	0.63**	–0.81**	0.62**	–0.59**	0.86**
11	0.61**	–0.82**	0.60**	–0.54**	0.78**
12	0.51*	–0.83**	0.62**	–0.53*	0.58**
13	0.38	–0.74**	0.64**	–0.59**	0.49*
14	0.32	–0.75**	0.63**	–0.63**	0.48*
15	0.26	–0.69**	0.56**	–0.62**	0.49*
16	0.25	–0.66**	0.55**	–0.66**	0.53*
17	0.26	–0.69**	0.54**	–0.65**	0.56**

** p < 0.0001 * p < 0.005.

Table 4 shows part of the results of a multiple linear regression analysis performed to predict the height of Japanese boys aged 5–17. The results for all ages are showed in S5 Table. The results show that a combination of weight and effective day length of the longest and shortest months is a significant predictor of height from childhood to adolescence. Both the effective day lengths of the longest and shortest months were negatively correlated with height. This means that if we control for weight, body height increases with shortening effective day length. The accuracy of the multiple regression model reached the maximum in early adolescence. Predictive power decreased and weight was no longer a significant predictor in late adolescence. The regression coefficient was greater for the day length of the shortest month than for the longest month throughout all ages.

**Table 4:** Regression coefficients (standard errors) of predictors for height (boys).

Boys	Predictors	Regression coefficient	Standard error	95% CI Lower	95% CI Upper	t	p	Adjusted R^2^

Age: 5	Weight	0.57	0.07	0.44	0.70	8.66	<0.001	0.876
	Longest5000	–0.36	0.09	–0.53	–0.18	–4.05	<0.001	
	Shortest5000	–0.75	0.09	–0.94	–0.57	–8.26	<0.001	
7	Weight	0.60	0.08	0.43	0.77	7.09	<0.001	0.839
	Longest5000	–0.43	0.10	–0.63	–0.22	–4.17	<0.001	
	Shortest5000	–0.73	0.11	–0.94	–0.51	–6.83	<0.001	
9	Weight	0.63	0.07	0.49	0.78	8.95	<0.001	0.890
	Longest5000	–0.49	0.08	–0.66	–0.31	–5.71	<0.001	
	Shortest5000	–0.77	0.09	–0.94	–0.59	–8.83	<0.001	
11	Weight	0.70	0.05	0.60	0.81	13.47	<0.001	0.932
	Longest5000	–0.36	0.07	–0.50	–0.22	–5.28	<0.001	
	Shortest5000	–0.66	0.07	–0.80	–0.53	–10.05	<0.001	
13	Weight	0.64	0.05	0.53	0.74	12.12	<0.001	0.933
	Longest5000	–0.44	0.07	–0.57	–0.30	–6.42	<0.001	
	Shortest5000	–0.79	0.07	–0.92	–0.66	–12.11	<0.001	
15	Weight	0.37	0.08	0.21	0.54	4.51	<0.001	0.826
	Longest5000	–0.65	0.10	–0.85	–0.44	–6.21	<0.001	
	Shortest5000	–1.06	0.11	–1.28	–0.84	–9.55	<0.001	
17	Weight	0.30	0.11	0.09	0.51	2.85	0.007	0.760
	Longest5000	–0.72	0.12	–0.97	–0.48	–5.92	<0.001	
	Shortest5000	–1.10	0.14	–1.37	–0.82	–8.09	<0.001	

Longest5000: Effective day length of the longest month at 5000 lux. Shortest5000: Effective day length of the shortest month at 5000 lux.

Table 5 shows part of the results of a multiple linear regression analysis performed to predict the height of Japanese girls aged 5–17. The results for all ages are shown in S5 Table. The results show that a combination of weight, effective day length of the longest and shortest months is a significant predictor of height from childhood to early adolescence. Both of effective day lengths of the longest and shortest months were negatively correlated with height. This means that if we control for weight, body height increases with shortening effective day length. The accuracy of the multiple regression model reached the maximum at 9 to 11 years of age, which was earlier than for boys. Predictive power decreased and weight was no longer a significant predictor in late adolescence. The regression coefficient was greater for the day length of the shortest month than for the longest month throughout all ages.

**Table 5:** Regression coefficients (standard errors) of predictors for height (girls).

Girls	Predictors	Regression coefficient	Standard error	95% CI Lower	95% CI Upper	T	p	Adjusted R^2^

Age: 5	Weight	0.61	0.06	0.48	0.74	9.81	<0.001	0.887
	Longest5000	–0.28	0.08	–0.45	–0.11	–3.36	0.002	
	Shortest5000	–0.67	0.09	–0.85	–0.49	–7.70	<0.001	
7	Weight	0.59	0.08	0.43	0.75	7.45	<0.001	0.854
	Longest5000	–0.36	0.10	–0.56	–0.17	–3.75	<0.001	
	Shortest5000	–0.71	0.10	–0.91	–0.50	–6.99	<0.001	
9	Weight	0.65	0.06	0.52	0.77	10.52	<0.001	0.910
	Longest5000	–0.36	0.08	–0.51	–0.20	–4.62	<0.001	
	Shortest5000	–0.69	0.08	–0.85	–0.54	–9.01	<0.001	
11	Weight	0.56	0.07	0.43	0.69	8.53	<0.001	0.881
	Longest5000	–0.38	0.09	–0.56	–0.19	–4.17	<0.001	
	Shortest5000	–0.82	0.09	–0.99	–0.64	–9.50	<0.001	
13	Weight	0.25	0.11	0.03	0.48	2.29	0.027	0.690
	Longest5000	–0.66	0.15	–0.96	–0.37	–4.53	<0.001	
	Shortest5000	–1.11	0.14	–1.39	–0.83	–7.95	<0.001	
15	Weight	0.19	0.10	–0.02	0.40	1.80	0.078	0.715
	Longest5000	–0.80	0.13	–1.07	–0.54	–6.04	<0.001	
	Shortest5000	–1.21	0.14	–1.49	–0.92	–8.58	<0.001	
17	Weight	0.23	0.11	0.01	0.44	2.11	0.040	0.714
	Longest5000	–0.77	0.13	–1.04	–0.51	–5.86	<0.001	
	Shortest5000	–1.15	0.15	–1.45	–0.85	–7.83	<0.001	

Longest5000: Effective day length of the longest month at 5000 lux. Shortest5000: Effective day length of the shortest month at 5000 lux.

Table 6 shows part of the results of a multiple linear regression analysis performed to predict the weight of Japanese boys aged 5–17. The results for all ages are shown in S5 Table. The results show that a combination of height, effective day length of the longest and shortest months is a significant predictor of weight from childhood to early adolescence. Both of the effective day lengths of the longest and shortest months were positively correlated with weight. This means that if height is controlled for, body weight increases with longer effective day length. The accuracy of the multiple regression model reached the maximum at 11 to 13 years of age. Predictive power decreased in late adolescence. The regression coefficient was greater for the day length of the shortest month than for the longest month before adolescence, which; however, reversed after puberty.

**Table 6:** Regression coefficients (standard errors) of predictors for weight (boys).

Boys	Predictors	Regression coefficient	Standard error	95% CI Lower	95% CI Upper	t	p	Adjusted R^2^

Age: 5	Height	1.11	0.13	0.85	1.36	8.66	<0.001	0.760
	Longest5000	0.47	0.12	0.21	0.72	3.73	<0.001	
	Shortest5000	0.68	0.18	0.32	1.03	3.85	<0.001	
7	Height	0.89	0.13	0.63	1.14	7.09	<0.001	0.762
	Longest5000	0.51	0.12	0.26	0.76	4.13	<0.001	
	Shortest5000	0.43	0.17	0.09	0.78	2.51	0.016	
9	Height	1.02	0.11	0.79	1.25	8.95	<0.001	0.824
	Longest5000	0.61	0.11	0.39	0.83	5.65	<0.001	
	Shortest5000	0.63	0.16	0.32	0.95	4.04	<0.001	
11	Height	1.14	0.08	0.97	1.32	13.47	<0.001	0.889
	Longest5000	0.49	0.08	0.33	0.66	5.97	<0.001	
	Shortest5000	0.71	0.11	0.48	0.93	6.42	<0.001	
13	Height	1.21	0.10	1.00	1.41	12.12	<0.001	0.874
	Longest5000	0.63	0.09	0.45	0.81	7.08	<0.001	
	Shortest5000	0.89	0.13	0.63	1.15	6.90	<0.001	
15	Height	0.85	0.19	0.47	1.23	4.51	<0.001	0.606
	Longest5000	0.67	0.19	0.29	1.05	3.52	0.001	
	Shortest5000	0.55	0.28	–0.01	1.12	1.97	0.055	
17	Height	0.52	0.18	0.15	0.88	2.85	0.007	0.588
	Longest5000	0.51	0.20	0.11	0.91	2.54	0.015	
	Shortest5000	0.08	0.28	–0.49	0.64	0.27	0.786	

Longest5000: Effective day length of the longest month at 5000 lux. Shortest5000: Effective day length of the shortest month at 5000 lux.

Table 7 shows part of the results of a multiple linear regression analysis performed to predict the weight of Japanese girls aged 5–17. The results for all ages are shown in S5 Table. The results show that a combination of height, effective day lengths of the longest and shortest months is a significant predictor of weight from childhood to early adolescence. Both of the effective day lengths of the longest and shortest months were positively correlated with weight. This means that if height is controlled for, body weight increases with longer effective day length. The accuracy of the multiple regression model reached the maximum at 9 to 11 years of age, which was earlier than the boys. Predictive power decreased in late adolescence. The regression coefficient was greater for the day length of the shortest month than for the longest month before adolescence, which however reversed after puberty.

**Table 7:** Regression coefficients (standard errors) of predictors for weight (girls).

Girls	Predictors	Regression coefficient	Standard error	95% CI Lower	95% CI Upper	t	P	Adjusted R^2^

Age: 5	Height	1.13	0.11	0.89	1.36	9.81	<0.001	0.792
	Longest5000	0.38	0.11	0.14	0.61	3.28	0.002	
	Shortest5000	0.62	0.15	0.31	0.93	4.01	<0.001	
7	Height	0.95	0.13	0.69	1.21	7.45	<0.001	0.764
	Longest5000	0.46	0.12	0.21	0.71	3.73	<0.001	
	Shortest5000	0.47	0.17	0.12	0.82	2.71	0.010	
9	Height	1.10	0.10	0.89	1.31	10.52	<0.001	0.847
	Longest5000	0.50	0.10	0.31	0.70	5.17	<0.001	
	Shortest5000	0.67	0.14	0.39	0.94	4.92	<0.001	
11	Height	1.11	0.13	0.85	1.37	8.53	<0.001	0.763
	Longest5000	0.59	0.12	0.35	0.84	4.88	<0.001	
	Shortest5000	0.84	0.17	0.50	1.18	4.96	<0.001	
13	Height	0.42	0.18	0.05	0.79	2.29	0.027	0.488
	Longest5000	0.68	0.20	0.28	1.09	3.37	0.002	
	Shortest5000	0.26	0.28	–0.30	0.82	0.93	0.356	
15	Height	0.37	0.20	–0.04	0.78	1.80	0.078	0.440
	Longest5000	0.46	0.24	–0.02	0.95	1.92	0.062	
	Shortest5000	0.00	0.32	–0.65	0.65	0.00	0.999	
17	Height	0.41	0.19	0.02	0.80	2.11	0.040	0.483
	Longest5000	0.37	0.23	–0.09	0.84	1.61	0.113	
	Shortest5000	–0.08	0.31	–0.70	0.54	–0.26	0.794	

Longest5000: Effective day length of the longest month at 5000 lux. Shortest5000: Effective day length of the shortest month at 5000 lux.

**Figure 3:**
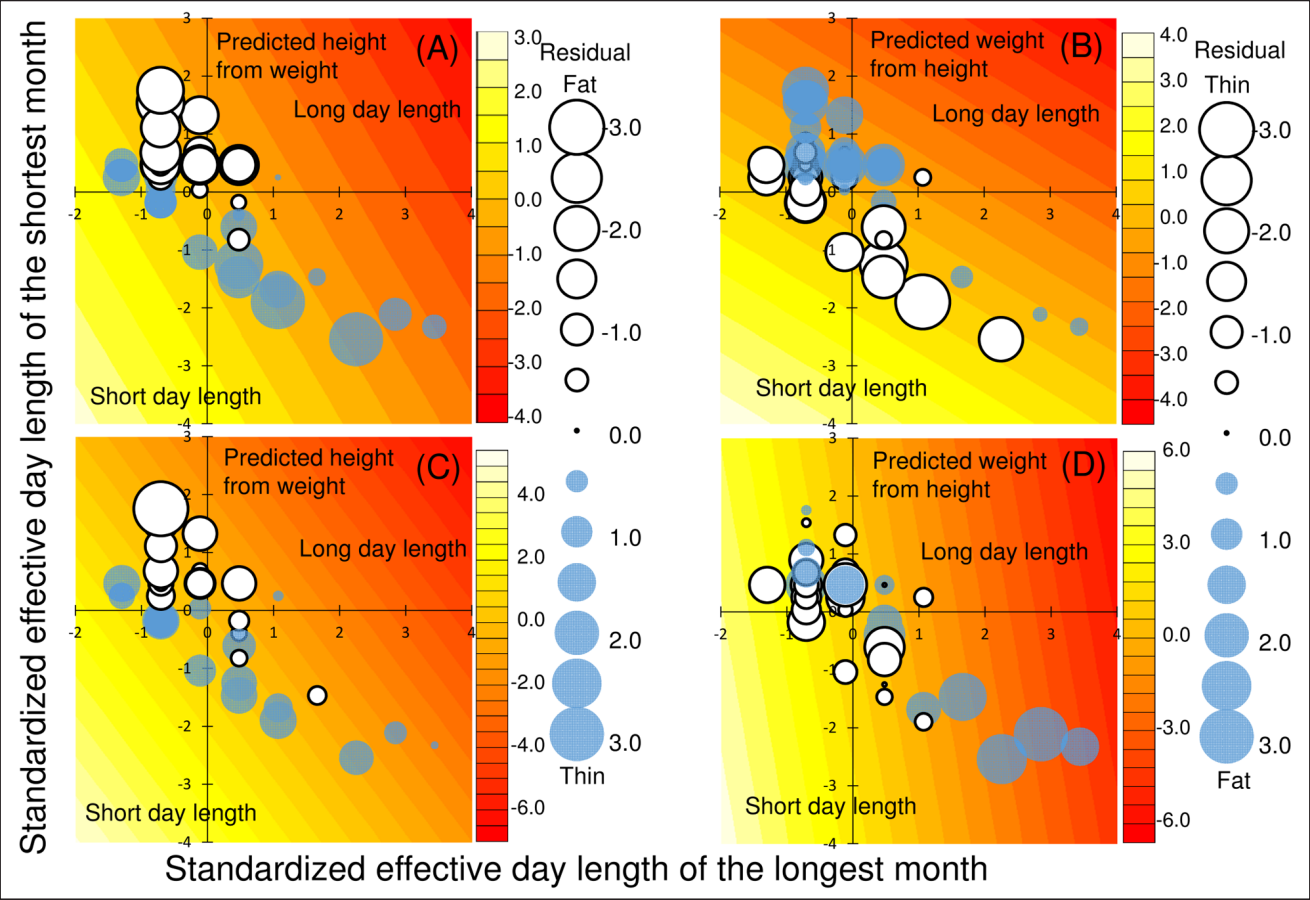
**Relationship between effective day length of the longest month and effective day length of the shortest month.** The relationship between effective day length of the longest shortest months for each prefecture, in relation to the results of multiple linear regression analysis performed to predict **(A)** height of 12-year-old boys, **(B)** weight of 12-year-old boys, **(C)** height of 17-year-old boys, and **(D)** weight of 17-year-old boys (Table S5). In relation to multiple regression models (5) and (6), the plot size was determined as the residual of (A) height minus first independent term (*H* – 0.69*W*), (B) weight minus first independent term (*W* – 1.13*H*), **(C)** height minus first independent term (*H* – 0.30*W*), and (D) weight minus first independent term (*W* – 0.52*H*). The gradation of the plot area indicates the sum of the values of the second independent term and the third independent term of multiple regression models (5) and (6), **(A)** – 0.35 *LONG* – 0.68 *SHORT*, **(B)** – (0.50 *LONG* + 0.72 *SHORT*), **(C)** – 0.72 *LONG* – 1.10 *SHORT*, **(D)** – (0.51 *LONG* + 0.08 *SHORT*). As the redness increased, the total effective day length in summer and winter increased.

Figure 3 shows the relationship between the effective day lengths of the longest and shortest months for each prefecture, in relation to the results of multiple linear regression analysis performed to predict (A) height of 12-year-old boys, (B) weight of 12-year-old boys, (C) height of 17-year-old boys, and (D) weight of 17-year-old boys. In relation to the multiple regression models (5) and (6), the plot size is shown as the value of (A) height minus first independent term (*H* – 0.69*W*), (B) weight minus first independent term (*W* – 1.13*H*), (C) height minus first independent term (*H* – 0.30*W*), and (D) weight minus first independent term (*W* – 0.52*H*). In (A) and (C), this value is the residual of height when controlled by weight and becomes small when body height is low in proportion to body weight, that is, in relatively fat individuals. In (B) and (D), this value is the residual of weight when controlled by height and becomes large when body weight is high in proportion to body height, that is, in relatively fat individuals. Moreover, the gradation of the plot area indicates the sum of the values of the second and third independent terms of multiple regression models (5) and (6), (A)–0.35 *LONG*–0.68 *SHORT*, (B) – (0.50 *LONG* + 0.72 *SHORT*), (C) – 0.72 *LONG* – 1.10 *SHORT*, and (D) – (0.51 *LONG* + 0.08 *SHORT*). As the redness increased, the total effective day length in summer and winter increased.

The result of multiple regression analysis shows that if weight is controlled for, body height decreases with longer effective day length. This means that the larger the sum of the values of the second and third independent variables, the more easily one gets fat. In addition, the result of multiple regression analysis shows that if height is controlled for, body weight increases with longer effective day length. This means that the larger the sum of values of the second and third independent variables, the more easily one gets fat.

The distribution of the plot in Figure 3 is a linear spread because a region with a long summer day length has a short winter day length and vice versa. In areas where long summer and winter day lengths coexist, the body composition becomes relatively short and fat. In areas where short summer and winter day lengths coexist, the body composition becomes relatively tall and thin. This tendency is clear in 12-year-old boys, but less so in 17-year-old boys. In estimating the weight of a 17-year-old boy, there is no clear relationship between weight difference and effective day length.

## Discussion

The characteristics of height and weight distribution of Japanese children and adolescents have something in common with the photoperiodic response of seasonal breeding vertebrates.

It has been reported that height and weight gain are often seasonal. It is generally said that healthy-weight children tend to increase in weight during winter and not so much during summer [28,29,30,31]. In contrast, there are many reports showing that height easily increases in summer [32,33,34,35,36,37].

On the other hand, many studies have reported that the geographical distribution of height is greater at higher latitudes [38,39,40], which is also true in Japan [1,2,3,4,5].

However, the question arises here. If the height is likely to grow in summer, the distribution seems to be similar to the distribution of summer day length. However, the height of Japanese children is greater in regions where the day lengths are shorter in winter than they are longer in summer [6].

Despite many studies suggesting that height is likely to increase under long day lengths, the height distribution is taller in regions with shorter day lengths. To explain this inconsistency, the height growth velocity in summer should be greater according to the shortness of the winter day length. In other words, the growth rate should be regulated by prior photoperiodic history. Such a mechanism for regulating physiological responses to the photoperiodic history is found in the photoperiodicity of seasonal breeding vertebrates [12,13,14,15,16].

Seasonal breeding vertebrates such as Siberian hamsters perform reproductive activities in anticipation of the arrival of spring according to shorter winter day length. The current model suggests that the melatonin right/dark signal sets the local circadian rhythm in the pituitary pars tuberalis (PT) by induction of clock genes [41,42,43]. This leads to activation of the thyroid-stimulating hormone β subunit (TSHβ) promoter in PT thyrotroph and promotes seasonal deiodinase signaling and thyroid hormone metabolism in tanycytes [12]. The two deiodinase enzyme genes (*DIO2* and *DIO3*) reciprocally regulate each other to determine the local concentration of triiodothyronine (T3), a biologically active form of thyroid hormone. These stimuli trigger the seasonality of the reproductive and immune systems [12,13,14,15,16].

These series of photoperiodic reactions are dependent on the prior photoperiodic history. These mechanisms are not yet fully understood; however, it is thought that epigenetic processes are involved, such as changes in chromatin accessibility to clock genes and their regulation [14], histone deacetylation in the hypothalamus [15], or DNA methylation status in the promoter region of deiodinase enzyme genes [17,44,45]. That is, it is considered that the photoperiodic history is preserved by the epigenetic change of the gene, and the expression is controlled according to the degree of the prior photoperiod. If the function is applicable to humans, summer height growth may quantitatively increase with winter day length, and height growth seasonality can be explained by the effect of thyroid hormone signaling [5].

In a previous report, the authors hypothesized that effective day length and thyroid hormone signaling could explain the geographical differences in height and weight in an integrated way. The distribution of height in Japanese early adolescence negatively correlated with annual mean effective day length. On the other hand, weight distribution, when controlled by height, positively correlated with annual mean effective day length. Assuming that annual mean effective day length was negatively related to thyroid hormone signaling, thyroid hormone was activated in areas where the effective day length was short, and it was thought that height would easily increase, unlike weight. Conversely, in areas where the effective day length was long, thyroid hormones were inactivated, and it was thought that height would hardly increase, as would the weight. [5].

On the other hand, photoperiodicity consists of two phases in which *DIO2* and *DIO3* were reciprocally activated and inactivated on short and long day lengths throughout the year [12,13,14,15,16]. This indicates that the geographical differences in physique distribution should essentially be explained by the summer and winter day length rather than the annual mean day length.

Even in this study, the multiple linear regression analysis to predict height, weight, and the effective day lengths of both the longest and shortest months was a significant predictor, and the prediction was the most robust in early adolescence. Without obesity and thinness, the relationship between height and weight becomes proportional, and effective day length should not be a significant predictor. Equations (5) and (6) are relationship explanatory variables, and objective variables are replaced with each other, so both the height and weight should be oppositely affected by the effective day length in order to become significant formulas. It is extremely rare in terms of probability that formulas (5) and (6) are significant at the same time without any special reason. In other words, effective day length negatively affects height (when weight is controlled), positively affects weight (when height is controlled), and seems to cause a deviation in the proportional relationship between height and weight.

In multiple regression analysis, both the effective day lengths of the longest (summer) and the shortest months (winter) were negative predictors of height in early adolescence. This seems to indicate that thyroid hormone is more activated in regions where the day length is short, irrespective of summer or winter. Conversely, in multiple regression analysis, the effective day lengths of both the longest (summer) and the shortest months (winter) were positive predictors for weight in early adolescence. This seems to indicate that thyroid hormone is more inactivated in regions where the day length is long, irrespective of summer or winter.

From these findings, it is considered that thyroid hormone signaling in certain regions is activated in the summer according to the prior winter photoperiodic history and inactivated in the winter according to the prior summer photoperiodic history. The results of multiple regression analysis suggest that the effects of winter and summer effective day lengths are not equivalent, and the winter day length seems to have a greater effect on thyroid hormone signaling. This is consistent with seasonal breeding vertebrates that switch between the two reproductive phases in summer and winter.

Here, from the viewpoint of obesity, the coexistence of relatively long summer and winter day lengths causes short and fat body composition because it will work negatively for an increase in height and positively for weight gain. However, the region that has a short day length in winter generally has a long day length in summer. For this reason, it does not simply mean that obesity is likely to occur in regions where the day length is long. Increased thyroid hormone signaling according to the short winter day length is thought to contribute mainly to height gain and weight loss. Decreased thyroid hormone signaling according to the long summer day length is thought to contribute mainly to height growth suppression and weight gain. Body composition is thought to change depending on the balance between summer and winter effective day lengths.

The prevalence rate of obesity in Japanese children and adolescents is higher on the Pacific side of northern and southern Japan [5]. In winter, the area on the Japan Sea side is affected by the northwestern monsoon and the weather is bad, thereby shortening the effective day length. In summer, the astronomically possible sunshine duration is longer in northern Japan, while the amount of solar radiation is greater in southern Japan, and the effective day length is the shortest in central Japan, near Tokyo. This may be the cause of the increased obesity prevalence on the Pacific side of northern and southern Japan [5].

Recently, the physiological pathway in which the photoperiodic environment causes obesity is gradually becoming apparent. *DIO2* regulates the calorific value of brown adipocytes, and it is becoming clear that the long-day environment leads to obesity [46,47,48,49,50,51,52,53]. Although this study is for healthy children, opinions are consistent that a long-day environment is likely to cause obesity.

On the other hand, little is known about the physiological mechanism of whether day length affects growth related to height. However, in recent years, it has been reported that the results of human growth hormone treatment differ depending on the latitude [34,35] and that it may be related to day length [37]. The activity of thyroid hormone signaling may be a potential cause of this phenomenon [5].

In this study, it was found that the geographical differences in physiques of Japanese children and adolescents can be described by the seasonal nature of the photoperiodic environment. This is consistent with seasonal breeding vertebrates switching their reproductive phases according to photoperiodic information. This phenomenon where the same explanatory variable is negatively correlated with height and positively correlated with weight, in a significant manner, is statistically rare, and there may be no physiological interpretation of this phenomenon other than one based on thyroid hormone activity. This time, a statistically more complex model showed that two explanatory variables (effective day lengths of the longest and shortest months) negatively correlated with height and positively correlated with weight at the same time, in a significant manner. This adds evidence to the previous hypothesis that the regional differences in physical constitutions of Japanese children occur as photoperiodic responses, and this further increases the possibility that geographical differences in physique and region-specific prevalence of obesity were caused by differences in photoperiodic environments.

The concept of effective day length and the association with thyroid hormone signaling may unitarily link geographical differences in physique with the relationship between day length and obesity at an individual level [5]. However, this study only explained part of the geographical differences in the proportion of healthy early adolescents. It is unclear whether a physiologically consistent explanation can be made after adolescence when sexual development becomes dominant. Certainly, the prevalence of obesity in Japanese children and adolescents is more common in areas where the proportion of physique is close to obesity as shown in this study, and these tendencies tend to be carried over to adults [54]. However, it is not certain whether the photoperiodic environment has effects even after puberty.

Additionally, it has been reported that obese children gain weight in summer due to circadian rhythm disturbances [55,56,57,58]; however, the increase in the weight of healthy children in areas with long day length is not a pathological condition. It is not clear whether day length itself causes morbid obesity.

In addition, there is an individual difference in whether or not humans show seasonality of weight gain or loss due to seasonal variations in day length, and the cause is unknown [59,60]. Whether humans show seasonal fluctuations in weight may have an important meaning as an indicator for assessing individual health. Careful investigation is needed to determine if natural daylight causes individual obesity.

All ecological studies can suffer from ecological fallacies, and this study only provides a partial explanation of the geographical differences in body size and proportions between Japanese children and adolescents. This study ignores the effects of regional differences in nutrient intake and genetic factors that cause regional differences in physique. However, these effects were not observed. It is clear that the improvement in physique achieved in history so far is due to the improvement of nutrient intake and improvement of the hygienic environment, and it must be taken into account that these effects are still present.

Furthermore, the physiological processes involved in this phenomenon must be evaluated in humans at the clinical level. In particular, there are many unclear points regarding the epigenetic mechanism in which the photoperiodic history is maintained, the role of the clock gene, and the pathway through which thyroid hormone signaling in the hypothalamus is involved in the regulation of height and weight. Therefore, additional studies using individual-level data to evaluate the impact of photoperiodic factors on anatomical factors should be pursued.

## Additional Files

The additional files for this article can be found as follows:

10.5334/jcr.198.s1 Supporting Information Figure S1. Map of the 47 prefectures of Japan The numbers correspond to the prefecture information presented in Tables S1–S4.

10.5334/jcr.198.s2 Supporting Information Table S1. Standardized height of 5- to 17-year-old children and adolescents in each prefecture of Japan averaged over a 25-year period (1989–2013).

10.5334/jcr.198.s3 Supporting Information Table S2. Standardized weight of 5- to 17-year-old children and adolescents in each prefecture of Japan averaged over a 25-year period (1989–2013).

10.5334/jcr.198.s4 Supporting Information Table S3. Basic association between standardized heights and weights for each age group.

10.5334/jcr.198.s5 Supporting Information Table S4. Population-weighted effective day length derived from Mesh Climatic Data 2000 for each prefecture of Japan.

10.5334/jcr.198.s6 Supporting Information Table S5. Results of a multiple linear regression analysis were performed to predict the height and weight of Japanese children and adolescents aged 5–17 years.
