# Supplementary figures and images for: Regional Differences in Height, Weight, and Body Composition may Result from Photoperiodic Responses: An Ecological Analysis of Japanese Children and Adolescents
Source: J Circadian Rhythms. 2021 Feb 22;19:3. doi: 10.5334/jcr.198 (PMC7908924; doi:10.5334/jcr.198)

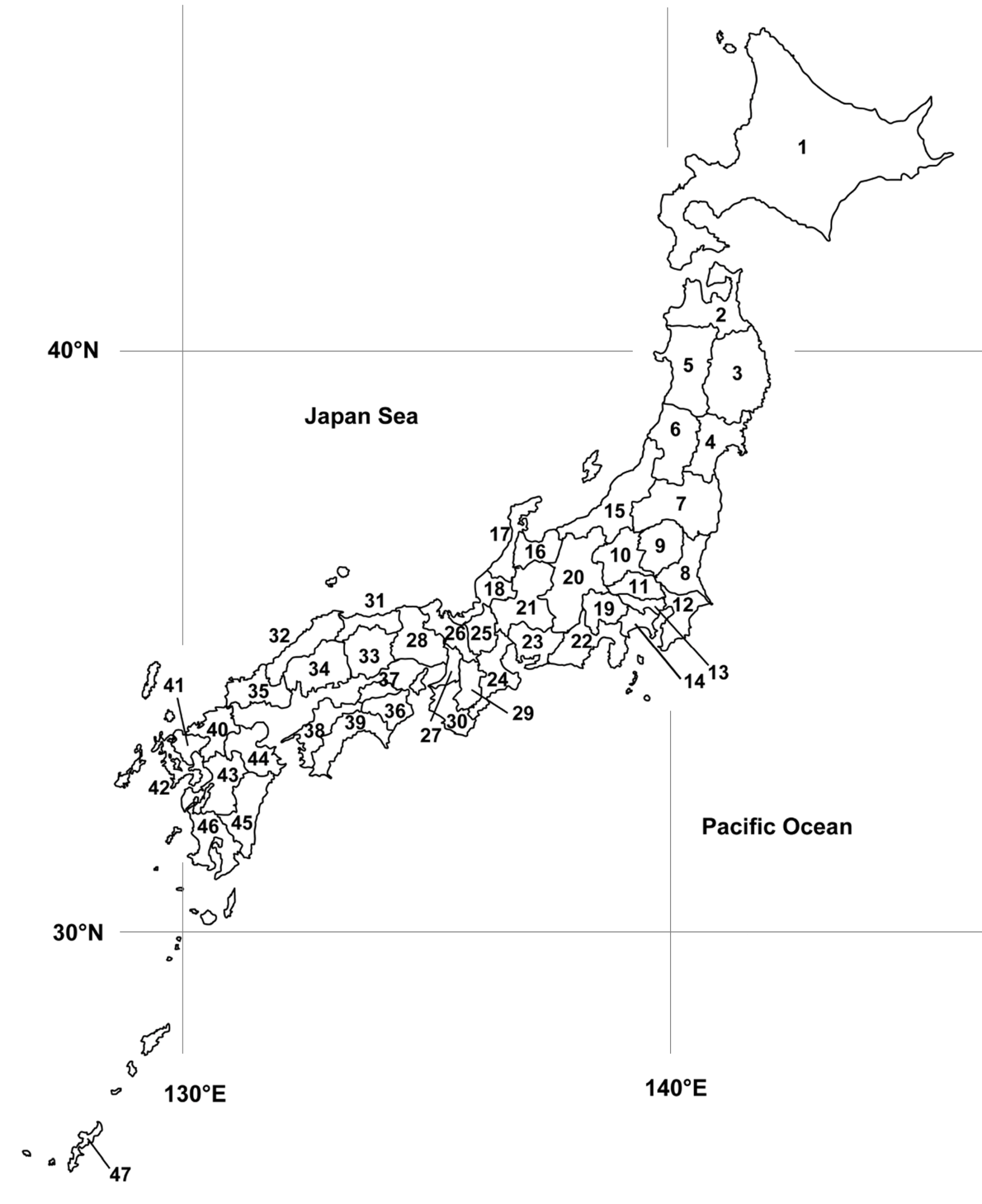

Supplement: Supporting Information Figure S1. — Map of the 47 prefectures of Japan The numbers correspond to the prefecture information presented in Tables S1–S4. [file jcr-19-198-s1.png]
